# (20*S*)-20-Acetamido-18-chloro-5α-pregnan-3β-yl acetate

**DOI:** 10.1107/S1600536810008883

**Published:** 2010-03-27

**Authors:** Michael Benn, Kanwal Nain Vohra, Masood Parvez

**Affiliations:** a Department of Chemistry, The University of Calgary, 2500 University Drive NW, Calgary, Alberta, Canada T2N 1N4

## Abstract

In the title compound, C_25_H_40_ClNO_3_, prepared by the thermolysis of (20*S*)-*O*,*N*-diacetyl-20-amino-*N*-chloro-3β-hydr­oxy-5α-pregnane, the three six-membered rings adopt chair conformations while the five-membered ring is in an envelope conformation. The ester group attached to ring *A* is in an equatorial position. All the rings are *trans*-fused. Intra­molecular C—H⋯O and C—H⋯Cl inter­actions occur. The crystal structure is stabilized by inter­molecular N—H⋯O and C—H⋯O inter­actions close contacts occur.

## Related literature

For background literature on the functionalization of the 18-methyl group of steroids, see: Pellissier & Santelli (2001 ▶). For the thermolysis of *N*-chloro­amides to achieve remote-site functionalizations, see: Edwards *et al.* (1971 ▶); Benn & Vohra, (1976 ▶); Vohra (1973 ▶). For bond-length data, see: Allen *et al.* (1987 ▶). For puckering parameters, see: Cremer & Pople (1975 ▶). For the preparation of (20*S*)-20-acetamido-3β-acet­oxy-5α-pregnane, see: Rej *et al.* (1976 ▶).
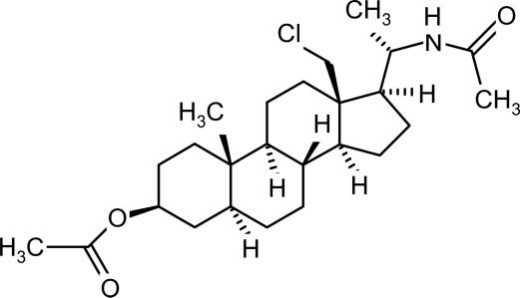


## Experimental

### 

#### Crystal data

C_25_H_40_ClNO_3_
*M* *_r_* = 438.03
Monoclinic, 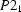

*a* = 7.6604 (4) Å
*b* = 9.7796 (4) Å
*c* = 16.8301 (8) Å
β = 96.398 (2)°
*V* = 1252.98 (10) Å^3^
*Z* = 2
Mo *K*α radiation
μ = 0.18 mm^−1^
*T* = 173 K
0.28 × 0.12 × 0.04 mm

#### Data collection

Nonius diffractometer with Bruker APEXII CCD detector
Absorption correction: multi-scan (*SORTAV*; Blessing, 1997 ▶) *T*_min_ = 0.952, *T*_max_ = 0.993
9843 measured reflections
5254 independent reflections
4999 reflections with *I* > 2σ(*I*)
*R*_int_ = 0.035

#### Refinement

*R*[*F* ^2^ > 2σ(*F* ^2^)] = 0.057
*wR*(*F* ^2^) = 0.154
*S* = 1.13
5254 reflections
275 parameters
1 restraint
H-atom parameters constrained
Δρ_max_ = 0.74 e Å^−3^
Δρ_min_ = −0.25 e Å^−3^
Absolute structure: Flack (1983 ▶), 2026 Friedel pairs
Flack parameter: 0.04 (9)

### 

Data collection: *COLLECT* (Hooft, 1998 ▶); cell refinement: *DENZO* (Otwinowski & Minor, 1997 ▶); data reduction: *SCALEPACK* (Otwinowski & Minor, 1997 ▶); program(s) used to solve structure: *SHELXS97* (Sheldrick, 2008 ▶); program(s) used to refine structure: *SHELXL97* (Sheldrick, 2008 ▶); molecular graphics: *ORTEP-3 for Windows* (Farrugia, 1997 ▶); software used to prepare material for publication: *SHELXL97*.

## Supplementary Material

Crystal structure: contains datablocks global, I. DOI: 10.1107/S1600536810008883/fb2184sup1.cif

Structure factors: contains datablocks I. DOI: 10.1107/S1600536810008883/fb2184Isup2.hkl

Additional supplementary materials:  crystallographic information; 3D view; checkCIF report

## Figures and Tables

**Table 1:** Hydrogen-bond geometry (Å, °)

*D*—H⋯*A*	*D*—H	H⋯*A*	*D*⋯*A*	*D*—H⋯*A*
N1—H1⋯O1^i^	0.88	2.03	2.893 (4)	165
C23—H23*C*⋯O3^ii^	0.98	2.54	3.487 (6)	163
C12—H12*B*⋯Cl1	0.99	2.62	3.076 (3)	108
C20—H20⋯Cl1	1.00	2.67	3.349 (3)	125
C20—H20⋯O1	1.00	2.42	2.812 (4)	103

Symmetry codes: (i) 
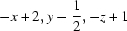
; (ii) 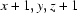.

**Fig. 1.**
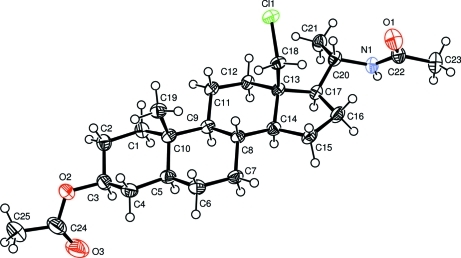
The title molecule with the displacement ellipsoids plotted at 50% probability level (Farrugia, 1997).

**Fig. 2.**
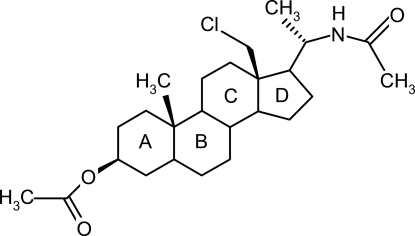
Lettering of the rings A—D of the title molecule.

**Fig. 3.**
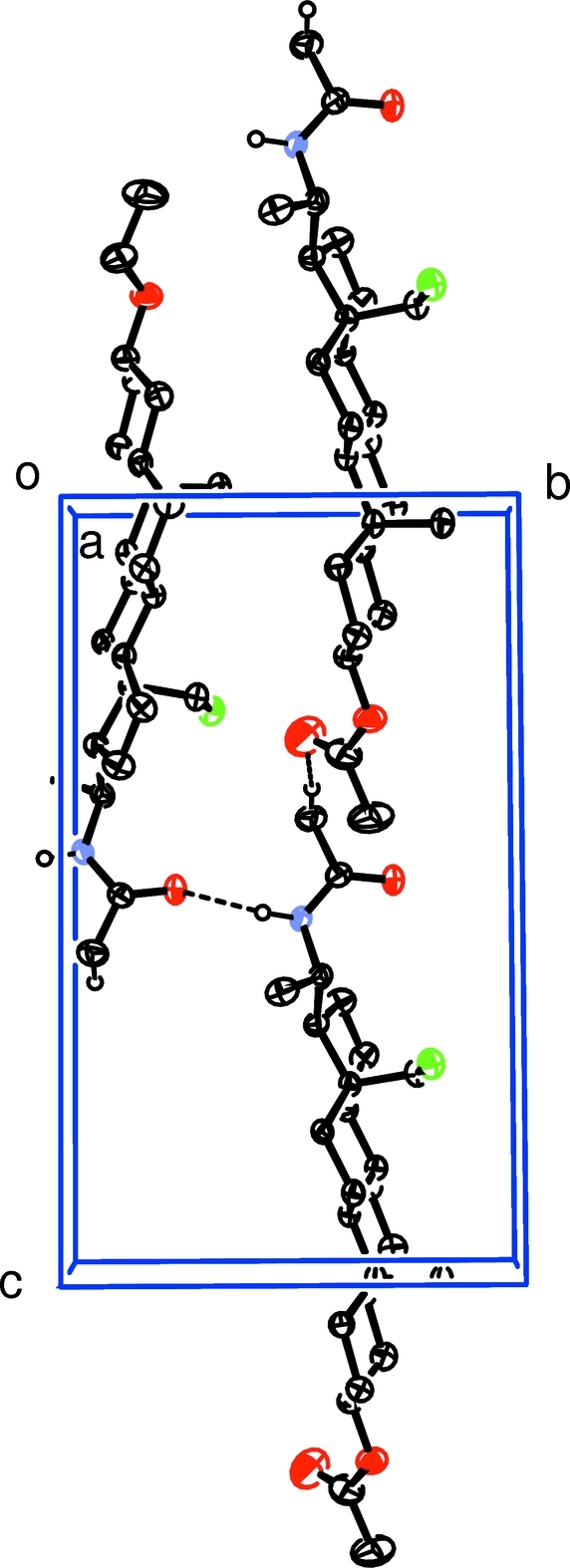
Unit cell packing showing hydrogen bonding interactions by dashed lines; the H-atoms not involved in H-bonds have been excluded for clarity.

## supplementary crystallographic information

### Comment

As an addition to the methods for the functionalisation of the 18-methyl group of the steroid system (Pellissier & Santelli, 2001) we utilized the thermolysis of an *N*-chloroamide: a procedure based on the known preference for a six-membered transition state in the abstraction of a hydrogen atom by a thermally generated N-centred amidyl radical (Edwards *et al.*, 1971). In this paper, we report the preparation, crystal structure and absolute configuration of the title compound prepared by the thermolysis of *N*-chloro-*O*, *N*-diacetyl-20*S*-amino-3β-hydroxy-5α-pregnane.

The title molecule is presented in Fig. 1. The molecule contains three six-membered rings A, B and C and a five-membered ring D (Fig. 2). All the rings are *trans*-fused. The rings A—C adopt chair conformations. The puckering parameters (Cremer & Pople, 1975) for the rings A to C are: *Q* = 0.581 (4), 0.579 (3), 0.581 (3) Å, θ = 1.6 (4), 3.5 (3), 0.0 (3)° and φ = 259 (23), 333 (7), 182 (15) °, respectively. The ring D adopts an envelope conformation with C13 being 0.703 (5) Å out of the mean-plane formed by the remaining ring atoms. The ester group attached to the ring A is in equatorial position. The bond lengths and angles are as expected (Allen *et al.*, 1987). There are intermolecular N—H···O and C—H···O hydrogen bonds. In addition, short intramolecular interactions involving Cl1 and O1 are also present in the structure; details have been provided in Tab. 1 and Fig. 3.

### Experimental

A solution of (20*S*)-20-acetamido-3β-acetoxy-5α-pregnane (Rej *et al.*, 1976) (500 mg) in CHCl_3_ was treated overnight with excess of *tert*-butyl hypochlorite, and the solvent and excess reagent were removed under reduced pressure (Rotovap, bath 313 K). The residual *N*-chloroamide was dissolved in aqueous 1,4-dioxane (1:4 v/v, 50 ml) containing dibenzoyl peroxide (20 mg) and calcium carbonate (2.5 g). The solution was boiled under reflux until a test for the *N*-chloro compound (moist starch/KI paper) was negative (ca. 2.5 h). The reaction mixture was cooled to room temperature, filtered, and the filter cake washed with CHCl_3_. The filtrate and washings were evaporated under reduced pressure (Rotovap, bath 323 K) and the residue subjected to preparative thin layer chromatography (Merck silica gel 60 PF254, 2 mm × 20 cm × 1 m), with CHCl_3_—MeOH (9:1 v/v) as eluent, and iodine for detection of the components. Elution of a band *R*_f_ 0.60 afforded (20*S*)-20-acetamido-3β-acetoxy-18-chloro-5α-pregnane (186 mg, 37%) which crystallized from ethanol-CHCl_3_ (ca. 7:1 v/v) in the form of colorless plates of average size 0.25 × 0.15 × 0.04 mm, m.p. 504-505 K (Leitz, uncorr.).

### Refinement

An absolute structure was established using anomalous scattering effects; 2026 Friedel pairs were measured. Though the H-atoms were observable in the difference electron density maps, they were included at geometrically idealized positions with N—H = 0.88 Å and C—H distances = 0.98, 0.99 and 1.00 Å for methyl, methylene and methine type H-atoms, respectively. The H-atoms were assigned U_iso_ = 1.2U_eq_ of the atoms to which they were bonded. The final difference map was free of chemically significant features.

^1^H-NMR (400 MHz, CDCl_3_  ref. res. H  δ_H_ 7.25 ppm) δ^H^  5.30 (1H, br d, *J* = 9.1 Hz), 4.63 (1H, m) 3.60 (1H, d, J =11.9 Hz), 3.49 (1H, d, *J* = 11.9 Hz), 1.99 (3H, s), 1.91 (3H, s), 1.23 (3H, d, *J* = 6.3 Hz) and 0.78 (3H, s); ^13^C-NMR (100 MHz, CDCl_3,_ ref  77.0 ppm) δ^C^ 170.7 s, 168.7 s, 73.5 d, 57.5 d, 57.0 d, 54.0 d, 45.9 s, 44.6 d, 45.5 t, 36.6 t, 35.7 d, 35.4 s, 35.0 s, 33.8 t, 31.7 t, 28.3 t, 27.3 t, 26.2 t, 23.4 q, 23.3 t, 22.2 q, 21.4 q, 20.7 t, 12.2 q; LRCIMS (NH_3_) m/z  438 (100) and 440 (30) (M+1 ^35^Cl and ^37^Cl resp.).

### Crystal data

**Table:**

C_25_H_40_ClNO_3_	*F*(000) = 476
*M**_r_* = 438.03	*D*_x_ = 1.161 Mg m^−^^3^
Monoclinic, *P*2_1_	Melting point = 504–505 K
Hall symbol: P 2yb	Mo *K*α radiation, λ = 0.71073 Å
*a* = 7.6604 (4) Å	Cell parameters from 2542 reflections
*b* = 9.7796 (4) Å	θ = 1.0–27.5°
*c* = 16.8301 (8) Å	µ = 0.18 mm^−^^1^
β = 96.398 (2)°	*T* = 173 K
*V* = 1252.98 (10) Å^3^	Plate, colorless
*Z* = 2	0.28 × 0.12 × 0.04 mm

### Data collection

**Table:**

Nonius diffractometer with Bruker APEXII CCD detector	5254 independent reflections
Radiation source: fine-focus sealed tube	4999 reflections with *I* > 2σ(*I*)
graphite	*R*_int_ = 0.035
φ and ω scans	θ_max_ = 27.5°, θ_min_ = 2.4°
Absorption correction: multi-scan (*SORTAV*; Blessing, 1997)	*h* = −9→9
*T*_min_ = 0.952, *T*_max_ = 0.993	*k* = −12→11
9843 measured reflections	*l* = −21→21

### Refinement

**Table:**

Refinement on *F*^2^	Secondary atom site location: difference Fourier map
Least-squares matrix: full	Hydrogen site location: difference Fourier map
*R*[*F*^2^ > 2σ(*F*^2^)] = 0.057	H-atom parameters constrained
*wR*(*F*^2^) = 0.154	*w* = 1/[σ^2^(*F*_o_^2^) + (0.0472*P*)^2^ + 1.6304*P*] where *P* = (*F*_o_^2^ + 2*F*_c_^2^)/3
*S* = 1.13	(Δ/σ)_max_ = 0.002
5254 reflections	Δρ_max_ = 0.74 e Å^−^^3^
275 parameters	Δρ_min_ = −0.25 e Å^−^^3^
1 restraint	Absolute structure: Flack (1983), 2026 Friedel pairs
144 constraints	Flack parameter: 0.04 (9)
Primary atom site location: structure-invariant direct methods	

### Special details

**Table:**

Experimental. ^1^H-NMR (400 MHz, CDCl_3_ ref. res. H δ_H_ 7.25 ppm) δ^H^ 5.30 (1H, br d, *J* = 9.1 Hz), 4.63 (1H, m) 3.60 (1H, d, J =11.9 Hz), 3.49 (1H, d, *J* = 11.9 Hz), 1.99 (3H, s), 1.91 (3H, s), 1.23 (3H, d, *J* = 6.3 Hz) and 0.78 (3H, s); ^13^C-NMR (100 MHz, CDCl_3,_ ref 77.0 ppm) δ^C^ 170.7 s, 168.7 s, 73.5 d, 57.5 d, 57.0 d, 54.0 d, 45.9 s, 44.6 d, 45.5 t, 36.6 t, 35.7 d, 35.4 s, 35.0 s, 33.8 t, 31.7 t, 28.3 t, 27.3 t, 26.2 t, 23.4 q, 23.3 t, 22.2 q, 21.4 q, 20.7 t, 12.2 q; LRCIMS (NH_3_) m/z 438 (100) and 440 (30) (M+1 ^35^Cl and ^37^Cl resp.).
Geometry. All esds (except the esd in the dihedral angle between two l.s. planes) are estimated using the full covariance matrix. The cell esds are taken into account individually in the estimation of esds in distances, angles and torsion angles; correlations between esds in cell parameters are only used when they are defined by crystal symmetry. An approximate (isotropic) treatment of cell esds is used for estimating esds involving l.s. planes.
Refinement. The following three reflections in the low angle range were deemed to be obstructed by the beam stop and were omitted: 1 1 0, -1 -1 1, 1 0 1

### Fractional atomic coordinates and isotropic or equivalent isotropic displacement parameters (Å^2^)

**Table:**

	*x*	*y*	*z*	*U*_iso_*/*U*_eq_	
Cl1	1.18745 (9)	0.31045 (8)	0.26306 (5)	0.03388 (19)	
O1	0.9823 (4)	0.2298 (2)	0.50569 (17)	0.0451 (7)	
O2	0.5711 (3)	0.1751 (3)	−0.28266 (16)	0.0413 (6)	
O3	0.3432 (5)	0.0304 (4)	−0.3034 (2)	0.0787 (12)	
N1	0.9983 (4)	0.0176 (3)	0.45452 (16)	0.0289 (6)	
H1	0.9844	−0.0703	0.4629	0.035*	
C1	0.8523 (4)	0.1056 (3)	−0.0897 (2)	0.0306 (7)	
H1A	0.9793	0.1224	−0.0749	0.037*	
H1B	0.8311	0.0065	−0.0840	0.037*	
C2	0.8040 (5)	0.1465 (4)	−0.1774 (2)	0.0344 (8)	
H2A	0.8354	0.2434	−0.1850	0.041*	
H2B	0.8708	0.0897	−0.2122	0.041*	
C3	0.6078 (5)	0.1265 (4)	−0.2005 (2)	0.0352 (8)	
H3	0.5792	0.0269	−0.1985	0.042*	
C4	0.4980 (4)	0.2047 (4)	−0.1458 (2)	0.0338 (7)	
H4A	0.3718	0.1854	−0.1610	0.041*	
H4B	0.5170	0.3041	−0.1515	0.041*	
C5	0.5500 (4)	0.1618 (3)	−0.0587 (2)	0.0295 (7)	
H5	0.5291	0.0610	−0.0562	0.035*	
C6	0.4329 (4)	0.2282 (4)	−0.0015 (2)	0.0355 (8)	
H6A	0.4516	0.3284	−0.0009	0.043*	
H6B	0.3081	0.2106	−0.0208	0.043*	
C7	0.4737 (4)	0.1720 (4)	0.0828 (2)	0.0315 (7)	
H7A	0.4423	0.0738	0.0830	0.038*	
H7B	0.4009	0.2203	0.1189	0.038*	
C8	0.6672 (4)	0.1886 (3)	0.11419 (19)	0.0246 (6)	
H8	0.6944	0.2883	0.1200	0.030*	
C9	0.7875 (4)	0.1252 (3)	0.0557 (2)	0.0260 (6)	
H9	0.7579	0.0256	0.0521	0.031*	
C10	0.7475 (4)	0.1843 (3)	−0.0313 (2)	0.0268 (6)	
C11	0.9805 (4)	0.1340 (3)	0.0888 (2)	0.0255 (6)	
H11A	1.0156	0.2313	0.0939	0.031*	
H11B	1.0528	0.0901	0.0508	0.031*	
C12	1.0170 (4)	0.0640 (3)	0.17103 (19)	0.0250 (6)	
H12A	0.9898	−0.0347	0.1655	0.030*	
H12B	1.1431	0.0734	0.1908	0.030*	
C13	0.9056 (4)	0.1278 (3)	0.23165 (19)	0.0233 (6)	
C14	0.7098 (4)	0.1199 (3)	0.1952 (2)	0.0276 (6)	
H14	0.6841	0.0206	0.1861	0.033*	
C15	0.6082 (4)	0.1624 (4)	0.2641 (2)	0.0336 (8)	
H15A	0.4895	0.1211	0.2584	0.040*	
H15B	0.5967	0.2631	0.2665	0.040*	
C16	0.7211 (4)	0.1070 (4)	0.3397 (2)	0.0365 (8)	
H16A	0.7466	0.1811	0.3793	0.044*	
H16B	0.6577	0.0330	0.3646	0.044*	
C17	0.8935 (4)	0.0516 (3)	0.3123 (2)	0.0281 (7)	
H17	0.8746	−0.0474	0.2994	0.034*	
C18	0.9533 (4)	0.2805 (3)	0.2470 (2)	0.0271 (7)	
H18A	0.8994	0.3123	0.2945	0.032*	
H18B	0.9027	0.3353	0.2006	0.032*	
C19	0.7999 (4)	0.3366 (3)	−0.0322 (2)	0.0318 (7)	
H19A	0.7475	0.3852	0.0103	0.038*	
H19B	0.7573	0.3767	−0.0841	0.038*	
H19C	0.9281	0.3447	−0.0232	0.038*	
C20	1.0498 (4)	0.0609 (3)	0.3773 (2)	0.0297 (7)	
H20	1.0877	0.1587	0.3821	0.036*	
C21	1.2082 (5)	−0.0250 (4)	0.3594 (2)	0.0396 (9)	
H21A	1.2517	0.0086	0.3104	0.047*	
H21B	1.1726	−0.1208	0.3523	0.047*	
H21C	1.3014	−0.0175	0.4041	0.047*	
C22	0.9714 (5)	0.1050 (4)	0.5129 (2)	0.0334 (7)	
C23	0.9265 (6)	0.0419 (5)	0.5896 (2)	0.0460 (10)	
H23A	0.8899	−0.0532	0.5798	0.055*	
H23B	0.8306	0.0934	0.6094	0.055*	
H23C	1.0300	0.0443	0.6295	0.055*	
C24	0.4371 (6)	0.1165 (5)	−0.3278 (3)	0.0534 (11)	
C25	0.4187 (7)	0.1767 (7)	−0.4111 (3)	0.0714 (16)	
H25A	0.4139	0.1028	−0.4506	0.086*	
H25B	0.5198	0.2356	−0.4172	0.086*	
H25C	0.3105	0.2308	−0.4195	0.086*	

### Atomic displacement parameters (Å^2^)

**Table:**

	*U*^11^	*U*^22^	*U*^33^	*U*^12^	*U*^13^	*U*^23^
Cl1	0.0272 (3)	0.0328 (4)	0.0424 (4)	−0.0044 (3)	0.0075 (3)	−0.0014 (4)
O1	0.0725 (19)	0.0211 (12)	0.0414 (15)	0.0050 (12)	0.0058 (13)	−0.0005 (11)
O2	0.0416 (14)	0.0481 (15)	0.0322 (14)	−0.0072 (12)	−0.0041 (11)	0.0025 (12)
O3	0.078 (2)	0.076 (3)	0.075 (3)	−0.034 (2)	−0.025 (2)	0.016 (2)
N1	0.0400 (15)	0.0204 (13)	0.0274 (15)	0.0024 (11)	0.0087 (12)	0.0028 (11)
C1	0.0325 (16)	0.0300 (17)	0.0295 (17)	0.0045 (13)	0.0041 (13)	0.0004 (13)
C2	0.0395 (18)	0.0337 (19)	0.0302 (18)	0.0012 (14)	0.0052 (14)	−0.0020 (14)
C3	0.0412 (18)	0.0342 (18)	0.0291 (18)	−0.0052 (15)	−0.0005 (14)	0.0009 (14)
C4	0.0278 (15)	0.0346 (18)	0.0377 (19)	−0.0024 (13)	−0.0025 (13)	0.0016 (15)
C5	0.0278 (15)	0.0253 (16)	0.0349 (18)	−0.0028 (12)	0.0007 (13)	0.0012 (13)
C6	0.0275 (16)	0.040 (2)	0.038 (2)	0.0012 (14)	0.0013 (14)	0.0005 (15)
C7	0.0210 (14)	0.0382 (18)	0.0357 (18)	0.0002 (13)	0.0045 (12)	0.0027 (15)
C8	0.0215 (13)	0.0237 (15)	0.0290 (16)	0.0013 (11)	0.0042 (11)	0.0004 (13)
C9	0.0260 (14)	0.0209 (14)	0.0314 (17)	0.0007 (11)	0.0043 (12)	0.0009 (12)
C10	0.0272 (15)	0.0202 (14)	0.0335 (17)	−0.0005 (12)	0.0059 (12)	0.0011 (13)
C11	0.0216 (13)	0.0236 (15)	0.0325 (17)	0.0049 (11)	0.0085 (12)	0.0000 (12)
C12	0.0265 (14)	0.0217 (14)	0.0278 (16)	0.0046 (11)	0.0081 (12)	−0.0012 (12)
C13	0.0217 (13)	0.0215 (14)	0.0278 (16)	0.0025 (11)	0.0083 (11)	−0.0014 (12)
C14	0.0279 (15)	0.0248 (15)	0.0303 (17)	−0.0015 (12)	0.0049 (12)	−0.0007 (13)
C15	0.0240 (15)	0.040 (2)	0.038 (2)	0.0010 (13)	0.0108 (13)	0.0056 (16)
C16	0.0274 (16)	0.048 (2)	0.0365 (19)	0.0020 (14)	0.0144 (14)	0.0036 (16)
C17	0.0293 (15)	0.0286 (16)	0.0278 (16)	−0.0011 (12)	0.0091 (12)	0.0021 (13)
C18	0.0215 (13)	0.0248 (17)	0.0355 (17)	−0.0004 (11)	0.0059 (12)	−0.0022 (12)
C19	0.0335 (15)	0.0235 (17)	0.0384 (19)	−0.0033 (12)	0.0034 (13)	0.0011 (13)
C20	0.0329 (16)	0.0265 (16)	0.0310 (18)	−0.0011 (13)	0.0097 (13)	0.0036 (13)
C21	0.0321 (17)	0.052 (2)	0.036 (2)	0.0106 (16)	0.0084 (15)	0.0066 (17)
C22	0.0409 (19)	0.0315 (18)	0.0278 (18)	0.0038 (14)	0.0036 (14)	0.0037 (14)
C23	0.062 (3)	0.046 (2)	0.032 (2)	0.006 (2)	0.0153 (18)	0.0034 (17)
C24	0.053 (2)	0.061 (3)	0.042 (2)	−0.008 (2)	−0.0127 (19)	−0.005 (2)
C25	0.067 (3)	0.099 (4)	0.043 (3)	−0.011 (3)	−0.016 (2)	0.009 (3)

### Geometric parameters (Å, °)

**Table:**

Cl1—C18	1.808 (3)	C11—H11A	0.9900
O1—C22	1.230 (4)	C11—H11B	0.9900
O2—C24	1.336 (5)	C12—C13	1.534 (4)
O2—C3	1.459 (4)	C12—H12A	0.9900
O3—C24	1.208 (6)	C12—H12B	0.9900
N1—C22	1.335 (5)	C13—C18	1.552 (4)
N1—C20	1.462 (4)	C13—C14	1.558 (4)
N1—H1	0.8800	C13—C17	1.560 (4)
C1—C2	1.534 (5)	C14—C15	1.525 (5)
C1—C10	1.542 (4)	C14—H14	1.0000
C1—H1A	0.9900	C15—C16	1.554 (5)
C1—H1B	0.9900	C15—H15A	0.9900
C2—C3	1.522 (5)	C15—H15B	0.9900
C2—H2A	0.9900	C16—C17	1.544 (4)
C2—H2B	0.9900	C16—H16A	0.9900
C3—C4	1.522 (5)	C16—H16B	0.9900
C3—H3	1.0000	C17—C20	1.532 (5)
C4—C5	1.533 (5)	C17—H17	1.0000
C4—H4A	0.9900	C18—H18A	0.9900
C4—H4B	0.9900	C18—H18B	0.9900
C5—C6	1.531 (5)	C19—H19A	0.9800
C5—C10	1.547 (4)	C19—H19B	0.9800
C5—H5	1.0000	C19—H19C	0.9800
C6—C7	1.521 (5)	C20—C21	1.533 (5)
C6—H6A	0.9900	C20—H20	1.0000
C6—H6B	0.9900	C21—H21A	0.9800
C7—C8	1.526 (4)	C21—H21B	0.9800
C7—H7A	0.9900	C21—H21C	0.9800
C7—H7B	0.9900	C22—C23	1.505 (5)
C8—C14	1.521 (4)	C23—H23A	0.9800
C8—C9	1.550 (4)	C23—H23B	0.9800
C8—H8	1.0000	C23—H23C	0.9800
C9—C11	1.524 (4)	C24—C25	1.513 (7)
C9—C10	1.573 (5)	C25—H25A	0.9800
C9—H9	1.0000	C25—H25B	0.9800
C10—C19	1.543 (4)	C25—H25C	0.9800
C11—C12	1.541 (4)		
			
C24—O2—C3	117.0 (3)	C11—C12—H12B	109.4
C22—N1—C20	123.2 (3)	H12A—C12—H12B	108.0
C22—N1—H1	118.4	C12—C13—C18	111.4 (2)
C20—N1—H1	118.4	C12—C13—C14	107.5 (3)
C2—C1—C10	113.3 (3)	C18—C13—C14	108.2 (2)
C2—C1—H1A	108.9	C12—C13—C17	118.4 (3)
C10—C1—H1A	108.9	C18—C13—C17	110.5 (3)
C2—C1—H1B	108.9	C14—C13—C17	99.9 (2)
C10—C1—H1B	108.9	C8—C14—C15	119.0 (3)
H1A—C1—H1B	107.7	C8—C14—C13	115.6 (3)
C3—C2—C1	109.8 (3)	C15—C14—C13	103.7 (3)
C3—C2—H2A	109.7	C8—C14—H14	105.8
C1—C2—H2A	109.7	C15—C14—H14	105.8
C3—C2—H2B	109.7	C13—C14—H14	105.8
C1—C2—H2B	109.7	C14—C15—C16	104.1 (3)
H2A—C2—H2B	108.2	C14—C15—H15A	110.9
O2—C3—C4	110.4 (3)	C16—C15—H15A	110.9
O2—C3—C2	106.3 (3)	C14—C15—H15B	110.9
C4—C3—C2	112.2 (3)	C16—C15—H15B	110.9
O2—C3—H3	109.3	H15A—C15—H15B	109.0
C4—C3—H3	109.3	C17—C16—C15	107.1 (3)
C2—C3—H3	109.3	C17—C16—H16A	110.3
C3—C4—C5	109.8 (3)	C15—C16—H16A	110.3
C3—C4—H4A	109.7	C17—C16—H16B	110.3
C5—C4—H4A	109.7	C15—C16—H16B	110.3
C3—C4—H4B	109.7	H16A—C16—H16B	108.5
C5—C4—H4B	109.7	C20—C17—C16	113.1 (3)
H4A—C4—H4B	108.2	C20—C17—C13	118.4 (3)
C6—C5—C4	112.1 (3)	C16—C17—C13	103.2 (3)
C6—C5—C10	112.0 (3)	C20—C17—H17	107.2
C4—C5—C10	112.8 (3)	C16—C17—H17	107.2
C6—C5—H5	106.5	C13—C17—H17	107.2
C4—C5—H5	106.5	C13—C18—Cl1	113.1 (2)
C10—C5—H5	106.5	C13—C18—H18A	109.0
C7—C6—C5	111.0 (3)	Cl1—C18—H18A	109.0
C7—C6—H6A	109.4	C13—C18—H18B	109.0
C5—C6—H6A	109.4	Cl1—C18—H18B	109.0
C7—C6—H6B	109.4	H18A—C18—H18B	107.8
C5—C6—H6B	109.4	C10—C19—H19A	109.5
H6A—C6—H6B	108.0	C10—C19—H19B	109.5
C6—C7—C8	112.1 (3)	H19A—C19—H19B	109.5
C6—C7—H7A	109.2	C10—C19—H19C	109.5
C8—C7—H7A	109.2	H19A—C19—H19C	109.5
C6—C7—H7B	109.2	H19B—C19—H19C	109.5
C8—C7—H7B	109.2	N1—C20—C17	110.5 (3)
H7A—C7—H7B	107.9	N1—C20—C21	108.3 (3)
C14—C8—C7	111.5 (3)	C17—C20—C21	113.5 (3)
C14—C8—C9	108.0 (2)	N1—C20—H20	108.1
C7—C8—C9	111.1 (3)	C17—C20—H20	108.1
C14—C8—H8	108.7	C21—C20—H20	108.1
C7—C8—H8	108.7	C20—C21—H21A	109.5
C9—C8—H8	108.7	C20—C21—H21B	109.5
C11—C9—C8	111.5 (3)	H21A—C21—H21B	109.5
C11—C9—C10	113.6 (3)	C20—C21—H21C	109.5
C8—C9—C10	112.1 (2)	H21A—C21—H21C	109.5
C11—C9—H9	106.4	H21B—C21—H21C	109.5
C8—C9—H9	106.4	O1—C22—N1	123.0 (3)
C10—C9—H9	106.4	O1—C22—C23	121.0 (3)
C1—C10—C19	108.7 (3)	N1—C22—C23	115.9 (3)
C1—C10—C5	107.5 (3)	C22—C23—H23A	109.5
C19—C10—C5	112.4 (3)	C22—C23—H23B	109.5
C1—C10—C9	110.4 (3)	H23A—C23—H23B	109.5
C19—C10—C9	109.9 (3)	C22—C23—H23C	109.5
C5—C10—C9	107.9 (2)	H23A—C23—H23C	109.5
C9—C11—C12	112.0 (2)	H23B—C23—H23C	109.5
C9—C11—H11A	109.2	O3—C24—O2	123.7 (4)
C12—C11—H11A	109.2	O3—C24—C25	126.1 (4)
C9—C11—H11B	109.2	O2—C24—C25	110.2 (4)
C12—C11—H11B	109.2	C24—C25—H25A	109.5
H11A—C11—H11B	107.9	C24—C25—H25B	109.5
C13—C12—C11	110.9 (2)	H25A—C25—H25B	109.5
C13—C12—H12A	109.4	C24—C25—H25C	109.5
C11—C12—H12A	109.4	H25A—C25—H25C	109.5
C13—C12—H12B	109.4	H25B—C25—H25C	109.5
			
C10—C1—C2—C3	−56.6 (4)	C11—C12—C13—C14	55.1 (3)
C24—O2—C3—C4	−87.3 (4)	C11—C12—C13—C17	167.1 (3)
C24—O2—C3—C2	150.8 (4)	C7—C8—C14—C15	−57.0 (4)
C1—C2—C3—O2	176.6 (3)	C9—C8—C14—C15	−179.4 (3)
C1—C2—C3—C4	55.9 (4)	C7—C8—C14—C13	178.5 (3)
O2—C3—C4—C5	−174.9 (3)	C9—C8—C14—C13	56.2 (3)
C2—C3—C4—C5	−56.5 (4)	C12—C13—C14—C8	−57.2 (3)
C3—C4—C5—C6	−175.1 (3)	C18—C13—C14—C8	63.2 (3)
C3—C4—C5—C10	57.4 (4)	C17—C13—C14—C8	178.7 (3)
C4—C5—C6—C7	173.7 (3)	C12—C13—C14—C15	170.8 (3)
C10—C5—C6—C7	−58.5 (4)	C18—C13—C14—C15	−68.9 (3)
C5—C6—C7—C8	55.8 (4)	C17—C13—C14—C15	46.6 (3)
C6—C7—C8—C14	−174.6 (3)	C8—C14—C15—C16	−163.5 (3)
C6—C7—C8—C9	−54.0 (4)	C13—C14—C15—C16	−33.4 (3)
C14—C8—C9—C11	−54.2 (3)	C14—C15—C16—C17	7.1 (4)
C7—C8—C9—C11	−176.8 (3)	C15—C16—C17—C20	150.9 (3)
C14—C8—C9—C10	177.2 (2)	C15—C16—C17—C13	21.7 (4)
C7—C8—C9—C10	54.6 (3)	C12—C13—C17—C20	76.9 (4)
C2—C1—C10—C19	−66.1 (4)	C18—C13—C17—C20	−53.2 (4)
C2—C1—C10—C5	55.9 (4)	C14—C13—C17—C20	−167.0 (3)
C2—C1—C10—C9	173.3 (3)	C12—C13—C17—C16	−157.3 (3)
C6—C5—C10—C1	176.3 (3)	C18—C13—C17—C16	72.6 (3)
C4—C5—C10—C1	−56.1 (3)	C14—C13—C17—C16	−41.2 (3)
C6—C5—C10—C19	−64.0 (4)	C12—C13—C18—Cl1	−46.1 (3)
C4—C5—C10—C19	63.5 (4)	C14—C13—C18—Cl1	−164.0 (2)
C6—C5—C10—C9	57.3 (3)	C17—C13—C18—Cl1	87.6 (3)
C4—C5—C10—C9	−175.2 (3)	C22—N1—C20—C17	−104.7 (4)
C11—C9—C10—C1	59.9 (3)	C22—N1—C20—C21	130.3 (3)
C8—C9—C10—C1	−172.7 (3)	C16—C17—C20—N1	43.4 (4)
C11—C9—C10—C19	−60.1 (3)	C13—C17—C20—N1	164.2 (3)
C8—C9—C10—C19	67.4 (3)	C16—C17—C20—C21	165.3 (3)
C11—C9—C10—C5	177.0 (3)	C13—C17—C20—C21	−73.9 (4)
C8—C9—C10—C5	−55.5 (3)	C20—N1—C22—O1	2.3 (6)
C8—C9—C11—C12	57.1 (3)	C20—N1—C22—C23	−177.5 (3)
C10—C9—C11—C12	−175.1 (2)	C3—O2—C24—O3	2.7 (7)
C9—C11—C12—C13	−58.1 (3)	C3—O2—C24—C25	−179.1 (4)
C11—C12—C13—C18	−63.2 (3)		

### Hydrogen-bond geometry (Å, °)

**Table:**

*D*—H···*A*	*D*—H	H···*A*	*D*···*A*	*D*—H···*A*
N1—H1···O1^i^	0.88	2.03	2.893 (4)	165
C23—H23C···O3^ii^	0.98	2.54	3.487 (6)	163
C12—H12B···Cl1	0.99	2.62	3.076 (3)	108
C20—H20···Cl1	1.00	2.67	3.349 (3)	125
C20—H20···O1	1.00	2.42	2.812 (4)	103

Symmetry codes: (i) −*x*+2, *y*−1/2, −*z*+1; (ii) *x*+1, *y*, *z*+1.
